# Aberrant hydroxymethylation in promoter CpG regions of genes related to the cell cycle and apoptosis characterizes advanced chronic myeloid leukemia disease, poor imatinib respondents and poor survival

**DOI:** 10.1186/s12885-022-09481-9

**Published:** 2022-04-14

**Authors:** Sameer Ahmad Guru, Mamta Pervin Sumi, Abdul Rashid Mir, Mirza Masroor Ali Beg, Bidhan Chandra koner, Alpana Saxena

**Affiliations:** 1 Lurie Children’s Hospital and Stanley Manne Children’s Research Institute, Northwestern University, Chicago, IL USA; 2 Department of Biochemistry, Multidisciplinary Research Unit (MRU), Maulana Azad Medical College, New Delhi, India; 3 Department of Inflammation and Immunity, Lerner Research Institute, Cleve Land Clinic, OH Cleveland, USA; 4 Kingdom of Saudi Arabia, University of Tabuk, Tabuk, Saudi Arabia; 5 Faculty of Medicine and Center for Promotion of Medical Research, Faculty of Medical Sciences, Ala-Too International University, Bishek, Kyrgyzstan; 6 Department of Biochemistry, Hamdard Institute of Medical Science and Research (HIMSR), New Delhi, India

**Keywords:** Chronic Myeloid Leukaemia, Imatinib, Epigenetics, Promoter hypermethylation, Cell cycle and apoptosis related genes

## Abstract

**Background:**

There is strong evidence that disease progression, drug response and overall clinical outcomes of CML disease are not only decided by BCR/ABL1 oncoprotein but depend on accumulation of additional genetic and epigenetic aberrations. DNA hydroxymethylation is implicated in the development of variety of diseases. DNA hydroxymethylation in gene promoters plays important roles in disease progression, drug response and clinical outcome of various diseases. Therefore in this study, we aimed to explore the role of aberrant hydroxymethylation in promoter regions of different tumor suppressor genes in relation to CML disease progression, response to imatinib therapy and clinical outcome.

**Methods:**

We recruited 150 CML patients at different clinical stages of the disease. Patients were followed up for 48 months and haematological/molecular responses were analysed. Haematological response was analysed by peripheral blood smear. BCR/ABL1 specific TaqMan probe based qRT-PCR was used for assessing the molecular response of CML patients on imatinib therapy. Promoter hydroxymethylation of the genes was characterized using MS-PCR.

**Results:**

We observed that promoter hydroxymethylation of DAPK1, RIZ1, P16INK4A, RASSF1A and p14ARF^ARF^ genes characterize advanced CML disease and poor imatinib respondents. Although, cytokine signalling (SOCS1) gene was hypermethylated in advanced stages of CML and accumulated in patients with poor imatinib response, but the differences were not statistically significant. Moreover, we found hypermethylation of p14^ARF^, RASSF1 and p16^INK4A^ genes and cytokine signalling gene (SOCS1) significantly associated with poor overall survival of CML patients on imatinib therapy. The results of this study are in agreement of the role of aberrant DNA methylation of different tumor suppressor genes as potential biomarkers of CML disease progression, poor imatinib response and overall clinical outcome.

**Conclusion:**

In this study, we report that promoter hydroxymethylation of DAPK1, RIZ1, P16INK4A, RASSF1A and p14ARF^ARF^ genes is a characteristic feature of CML disease progressions, defines poor imatinib respondents and poor overall survival of CML patients to imatinib therapy.

**Supplementary Information:**

The online version contains supplementary material available at 10.1186/s12885-022-09481-9.

## Background

The importance of epigenetics in the pathogenesis of different human malignancies, especially leukaemias, has gained much recognition during the recent past [1, 2]. DNA methylation, in addition to playing a vital role in the development of leukaemia, also affects its progression and relapse by altering the expression patterns of various tumor suppressor and cell cycle regulating genes. Methylation of tumor suppressor gene p15, for example, is the most frequently reported epigenetic event that has been observed in myeloid malignancies. A number of other genes have been reported to be methylated in myeloid malignancies including in chronic myeloid leukaemia (CML) and acute myeloid leukaemia (AML), however the effects of these changes on the development, progression and relapse of disease are not completely understood [3, 4].

Chronic myeloid leukaemia is initiated with a single molecular event, the reciprocal translocation between chromosomes 9 and 22, t (9;22), which ultimately results in the formation BCR/ABL1 fusion protein with aberrant tyrosine kinase activity[5]. Although being genetically homogenous at the initial phase, a considerable genetic heterogeneity is seen in the clinical course of CML as it progresses at a varying rate from less aggressive chronic phase (CP) to more aggressive accelerated (AP) and blastic (BP) phases. Tyrosine kinase inhibitor; imatinib (Gleevec/STI-571, Novartis, Switzerland)- the first line treatment for CML patients, is very effective for treating CP-CML, however considerably poor outcomes are achieved in patients with advanced phase CML disease[5]. The heterogeneity in disease progression and drug (imatinib) response can be attributed to the molecular events including genetic and epigenetic viz, secondary mutations or additional chromosomal aberrations and CpG methylation patterns or histone modifications that follow BCR/ABL1 fusion [5–8]. In addition, BCR/ABL1 fusion protein enhances survival of hematopoietic stem cells and exert antiapoptotic activity in CML cell progenitors and mediating many of the processes like imatinib resistance, disease progression and response [9]. These effects of BCR/ABL1 are exerted by modulating the antiapoptotic genes like inducing Bcl-XL which is an antiapoptotic gene, blocking mitochondrial release of cytochrome C [10, 11] and also by inhibiting proapoptotic genes for example Bad or Bim [12, 13]. The mechanism of imatinib action is mediated by different pathways and Fas mediated apoptosis pathway plays an important role [14]. There are studies reporting that Fas-mediated apoptosis in CML is also operative in other treatment strategies like IFN-alpha where apoptosis follows Fas-R expression increase. Therefore, increasing Fas-R expression on LSCs increases their exposure to cytotoxic therapy like TKIs[15].

There are numerous studies which have focussed on elucidation of additional chromosomal abnormalities (ACAs) and other associated genetic events, considered as hallmarks of multistep disease progression in CML and a characteristic features of clonal evolution [16, 17]. There are reports that about 10% to 12% of CML patients show ACAs not only in advanced blastic phase but in chronic phase as well and appearance of these ACAs have the ability to cause different features in CML patients according to Ph pattern. Deletion of chromosome 9, for example is reported to be associated with bad survival outcome and monosomy of chromosome 7 is linked to the development of myeloid dysplastic syndrome (MDS) or acute myeloid leukaemia (AML) in CML patients having Ph-negative status. Further, ACAs have been associated with failure of CML management with imatinib therapy [18].

A few studies have examined the epigenetic events in association with CML disease progression and drug response[5, 5, 7]. However, these studies have been conducted with limited number of total patients with a further decrease in number of subjects at each clinical stage of the disease.

To address the issues of CML disease progression, drug resistance and poor overall clinical outcome with the existing therapies, the secondary molecular aberrations including both genetic and epigenetic, of which methylation of promoter regions of genes bears priority, must be explored. Therefore in this study, we determined the DNA methylation status at CpG dinucleotides of DAPK1, RASSF1, p16^INK4^, p14^ARF^, RIZ1 and SOCS1 in concert with disease progression, imatinib response and overall survival in chronic myelogenous leukaemia. We selected these six genes as majority of human malignancies have universal silencing of these genes, their inactivation/low levels have diagnostic and prognostic values in malignancies, hypermethylation of these genes (RASSF1 for example) has been implicated as initial events in carcinogenesis in general but rarely found in normal tissues, these genes have important roles in cell proliferation, cell cycle and apoptosis and as discussed above these processes affect imatinib response [19, 20]. Here, we report that aberrant promoter methylation of DAPK1, RIZ1, P16INK4A, RASSF1A and p14ARF^ARF^ genes is significantly associated with; CML disease progression to advanced clinical stages, poor imatinib response and poor overall survivals as well. We also report that SOCS1 (cytokine signalling) gene promoter methylation did not show any statistical association either with CML disease progression or imatinib response, but significantly associated with poor overall disease survival. Therefore, the results of our study indicate that hypermethylation at CpG dinucleotides of these genes is an important process of CML disease progression and characteristic feature of CML patients with poor imatinib response and poor overall survival.

## Methods

### Patients and healthy donors

The enrolled study population included a total of 150 CML patients in different clinical stages of CML disease and 150 age and gender matched healthy controls. Normal healthy controls were selected from a large pool individual who had no history of the malignancy or any other disease. Only Out of 150 CML patients, we included 100 CML patients who were in chronic phase (CP) and 25 each of accelerated phase (AP) and blast crisis (BC). Institutional Ethics committee (IEC) of Maulana Azad Medical College (MAMC) and associated hospitals approved the study. Peripheral blood/bone marrow samples were collected from the study subjects after written informed consents. The diagnosis of CML patients was made by clinical and haematological examination of peripheral blood/bone marrow. The CML diagnosis was confirmed by molecular detection of BCR/ABL1 fusion gene transcripts using multiplex reverse transcriptase polymerase chain reaction (multiplex RT-PCR) as detailed previously [21–23].

### Blood sampling and molecular detection of BCR/ABL1 fusion gene transcripts

We collected 4 mL of peripheral blood in Ethylenediaminetetraacetic acid (EDTA) anticoagulant vials. Peripheral Blood Leukocytes (PBLs) were isolated from 4 mL of blood collected in EDTA anticoagulant vials. RNA was extracted from PBLs through manual extraction method using the Trizol RNA extraction reagent (Amresco Life Sciences, USA). Prior to complimentary DNA (cDNA) synthesis RNA concentration and quality was assessed with an IMPLEN Nanophotometer (IMPLEN, INC. CA). Absorbance at 260 nm was measured for quantification of nucleic acid concentration. Purity of RNA was assessed by the absorbance ratio 260/280 and 260/230. Reverse transcription of the purified RNA was carried out using the Verso cDNA synthesis kit (Thermo Scientific, USA) according to manufacturer’s instructions. Briefly, the freshly purified RNA was first incubated at 72 °C for 10 min with random primers and nuclease free water. Following addition of the RNAse inhibitor, reverse transcriptase and cDNA synthesis master mix, the samples were incubated at 42 °C for 1 h and finally at 95 °C for 5 min to terminate the reaction. The cDNA was chilled at 4 °C for 10 min in the thermo cycler itself before transferring it to -80 °C storage for further analysis. The amount of RNA used was 200 ng/µL for each sample in cDNA synthesis.

Next the cDNA synthesized was used to amplify *BCR/ABL1* fusion gene transcripts using tetra primer multiplex RT-PCR. Tetra-primer multiplex RT-PCR system is able to detect different *BCR/ABL1* fusion gene transcript variants viz. b_2_/a_2_, b_3_/a_2_, e1a2. The primer sequences used for the detection of *BCR/ABL1* fusion gene transcripts were adopted as previously mentioned [24]. The primer sequences with annealing temperature used in this PCR system are given in Table 1. The original PCR thermal cycling profile was used with an annealing temperature of 64^◦^C as mentioned in Table 1. The healthy control subjects were confirmed to have normal CBC and negative for *BCR/ABL1* fusion gene transcripts.

**Table1:** Primers used in multiplex PCR for detection of BCR/ABL1 fusion gene transcripts [19]

Primer code	Primer Sequence	Annealing temperature
A	5′-ATAGGATCCTTTGCAACCGGGTCTGAA-3′	64^◦^C
B	5′-ACAGAATTCCGCTGACCATCAATAAG-3′	
C	5′-ACCGCATGTTCCGGGACAAAAG-3′	
D	5′-ACAGAATTCCGCTGACCATCAATAAG-3′	

## Patient follow-up and evaluation of molecular and hematologic response

The patients were followed-up for 48 months following imatinib mesylate (Gleevec/STI-571, Novartis, Switzerland) therapy. Examination of bone marrow biopsies/aspirates was undertaken at enrolment if indicated after assessing the clinical situation in detail. The diagnosis of different clinical stages of CML disease were made on the basis of the guidelines mentioned by European Leukaemia Net (ELN) and for demarcation of AP and BC stages of the disease, ELN criteria for AP (blast count 15–29% in peripheral blood or bone marrow) was used as AP defining event [25]. The imatinib dosages were administrated for the patients based on their hematologic and non-hematologic toxicities, ranging from 200 to 600 mg daily for CP, 300 mg to 800 mg for AP and 600 mg to 800 mg for BP. Patients were monitored after every fifteen days and drug response/toxicity evaluated after initiation of imatinib therapy.

The Complete Hematologic Response (CHR) was evaluated as defined by examining the patients for complete blood counts (WBC count below 10 × 10^9^/L), the absence of immature cells (blast cells, myelocytes and promyelocytes) using microscopic assessment of peripheral blood and other clinical features like splenomegaly. Moreover, molecular response was assessed once after the initiation of imatinib therapy. An undetectable BCR/ABL1 fusion gene transcripts was defined as; MR^4^, MR^4.5^ with BCR-ABL1 expression ≤ 0.01% and ≤ 0.0032% respectively compared to the base line levels, whereas a ≤ 0.1% reduction in BCR/ABL1 transcript titters compared to the base line levels was defined as Major Molecular Response (MMR). No Molecular Response (NMR) was also defined when an increasing change in BCR-ABL1/ ABL1 were detected.

## DNA isolation and analysis of methylation pattern at CpG dinucleotide sites of different genes

The samples were processed for isolation of DNA after confirming CML diagnosis by detection of *BCR/ABL1* fusion gene transcripts with multiplex RT-PCR. The isolation of DNA was carried out using commercially available DNA isolation kit (Gene Aid, India), according to the manufacturer’s instructions as previously described [26]. The quality and integrity of DNA was checked by 1% agarose gel stained with 0.5 μg/mL Ethidium Bromide (EtBr, stock 10 mg/mL), prepared in 1X Tris base, acetic acid and EDTA (TAE, pH-8.3) and quantified by NanoDrop spectrophotometer (Washington, DC, USA).

A standardized genomic DNA concentration (50 µg/µL) was modified with sodium bisulphite using EZ-DNA methylation kit (Zymo Research, India). Bisulphite-modified DNA samples were stored at -80 °C until used. The methylation of various genes at CpG dinucleotides of promoter regions was analysed using methylation specific polymerase chain reaction (MS-PCR). The primer sequences used for characterization of CpG methylation are mentioned in Table 2 below. The amplification program of MS-PCR consisted of 40 cycles with 95 °C as initial denaturation temperature and 40 cycles of denaturation at 95 °C for 45 s, annealing temperatures (specific for each gene), and extension of 72 °C. A final extension temperature of 72 °C was used for final amplification. The PCR amplicons were resolved on 2% EtBr stained agarose gel prepared in 1 × TAE buffer.

**Table 2:** Primers used to study promoter hypermethylation of different genes

Gene	Name	Primer sequence
DAPK1	Unmethylated forward	5′-GGAGGATAGTTGGATTGAGTTAATGTT-3′
	Unmethylated Reverse	5′-CAAATCCCTCCCAAACACCAA-3′
	Methylated forward	5′-GGATAGTCGGATCGAGTTAACGTC-3′
	Methylated Reverse	5′-CCCTCCCAAACGCCGA-3′
RIZ-1	Unmethylated forward	5′-TGGTGGTTATTGGGTGATGGT-3′
	Unmethylated Reverse	5′-ACTATTTCACCAACCCCAAGA-3′
	Methylated forward	5′-GTGGTGGTTATTGGGCGACGGC-3′
	Methylated Reverse	5′-GCTATTTCGCCGACCCCGACG-3′
P16INK4A	Unmethylated forward	5′-TTATTAGAGGGTGGGGTGGATTGT-3′
	Unmethylated Reverse	5′-CAACCCCAAACCACAACCATAA -3′
	Methylated forward	5′-TTATTAGAGGGTGGGGCGGATCGC-3′
	Methylated Reverse	5′-GACCCCGAACCGCGACCGTAA -3′
RASSF1A	Unmethylated forward	5′-GGGTTTTGCGAGAGCGCGT-3′
	Unmethylated Reverse	5′-GCTAACAAACGCGAACCG-3′
	Methylated forward	5′- GGTTTTGTGAGTGTGTTTAGT-3′
	Methylated Reverse	5′-CACTAACACACAAACCAAACA-3′
SOCS1	Unmethylated forward	5′-TCGTTCGTACGTCGATTATC-3′
	Unmethylated Reverse	5′-AAAAAAATACCCACGAACTCG-3′
	Methylated forward	5′-TATTTTGTTTGTATGTTGATTATTG-3′
	Methylated Reverse	5′-AAACTCAACACACAACCACTC-3′
p14ARF^ARF^	Unmethylated forward	5'-TTTTGGTGTTAAAGGGTGGTGTAGT-3 ‘
	Unmethylated Reverse	5'- CACAAAAACCCTCACTCACAACAA-3'
	Methylated forward	5'-GTGTTAAAGGGCGGCGTAGC-3’
	Methylated Reverse	5'- AAAACCCTCACTCGCGACGA-3'

## Statistical analysis and data interpretation

The differences among CML patients with different clinical stage and imatinib response groups at each CpG site were measured by the Fisher’s exact test to assess whether the differences existed varied significantly. Differences in survival of patients showing hypo-and hypermethylation at the CpG sites analysed in this study were calculated using Kaplan–Meier method. The comparisons that showed a *p*-value less than 0.05 were considered statistically significant. The statistical comparisons of categorical data were performed using GraphPad Prism 5 and survival comparisons were done using SPSS 20.0 software packages.

## Results

### Study population

The diagnosis of suspected CML patients who were presented at Lok Nayak Hospital, New Delhi, from February 2015 to January 2019, was confirmed by the molecular detection of *BCR/ABL1* fusion gene transcripts, as discussed in methodology section, in the Leukaemia Diagnosis Laboratory, Department of Biochemistry, Maulana Azad Medical College (MAMC) and Associated Hospitals, New Delhi. Atypical CML patients were excluded from the study. The final cohort included 150 CML patients in different clinical stages and 150 age and gender matched healthy controls. Demographic features and base line disease characteristics and haematological parameters of the study population are shown in Tables 3, 4 and 5 below.

**Table 3:** CML patients’ demographic features and imatinib dose

Age/Sex of CML patients	CML Patients (*n* = 150)	Imatinib 200 mg to 600 mg OD dose group (*n* = 59)	Imatinib 600 mg to 800 mg OD dose group (*n* = 91)
**Age (year) median (range)**			
All CML patients	38 (15–80)	38 (15–80)	40 (16–66)
Age ≤ 38 years	30 (15–38)	30 (15–38)	30 (16–38)
Age > 38 years	47 (40–80)	48 (40–80)	47 (40–66)
**Sex, n (%)**			
Male	89(59)	37 (41.5)	52 (58.5)
Female	61(41)	22 (36)	39 (64)

**Table 4:** Clinical and disease characteristics of CML patients at base line

Disease characteristics	CML Patients (*n* = 150)	Imatinib 400-mg OD dose group (*n* = 59)	Imatinib 600-mg OD dose group (*n* = 91)
**Splenomegaly, n (%)**			
No	57 (38)	27 (47)	30 (53)
Yes	93 (62)	32 (34)	61 (66)
**Hepatomegaly, n (%)**			
No	113 (75)	44 (39)	69 (61)
Yes	37 (25)	15 (40.5)	22(59.5)

**Table 5:** Haematological parameters of CML patients at base line

Characteristics	CML Patients (*n* = 150)	Imatinib 400-mg OD dose group (*n* = 59)	Imatinib 600-mg OD dose group (*n* = 91)
**Total Leukocyte Count/mm**^**3**^** (TLC)**			
Median	1,42,200	1,54,000	1,25,800
Range	3,460—9,64,000	19,520–9,64,000	3,460–6,73,650
**Hemoglobin (gm/dL)**			
Median	9.3	9	9.8
Range	4.7–17.2	4.7–13.4	5–17.2
**Platelets (Lacs/mm**^**3**^**)**			
Median	2.95	2.29	3.75
Range	1.78–15.11	1.79–2.97	1.78–15.11

### Assessment of haematological and molecular responses of CML patients to imatinib therapy

The CML patients (150) included in the study were followed for 48 months. Mean duration of follow- up was 23.7 ± 6.68 months and ranged from 3 to 48 months. During the follow-up of these 150 CML patients, complete haemogram (Hb, TLC, DLC, platelet count and ESR) was assessed at regular intervals. The haematological response to imatinib was analyzed to assess whether the patients achieved Complete Hematological Response (CHR) and the time to achieve CHR was noted (THR). Mean THR was 5.6 ± 5.02 months (range 1- 12 months). Each patient’s haematological response was based on patient’s best response during the course of treatment till 12^th^ month. Of the 100 chronic phase, 25 accelerated phase and 25 blast crisis CML patients 97 (97%), 21 (84%) and 17 (68%) achieved Complete Hematologic Response respectively (Table 6). Therefore, the rate of haematological response was more in case of CP-CML patients followed by AP-CML and then BC-CML patients. Moreover, the duration of achieving haematological response was lesser in patients with CP-CML disease (within 6 months of follow-up) than those patients with advanced stage (AP and BC) of the disease (between 8 to 12 months of the follow-up). Relapse of the disease was defined as increase in white blood count > 2000/mm^3^, platelet count ≥ 600,000/mm^3^, appearance of blasts in peripheral blood [27].

**Table 6:** Time taken for achievement of complete hematological response (CHR)

	**3 months**	**6 months**	**12 months**
No. of patients	150	150	150
CHR achieved, n (%)	97(65%)	104 (69%)	135 (90%)
CHR not achieved, n(%)	53(35%)	46(31%)	15 (10%)

Molecular Response (BCR-ABL1/ABL %) was assessed once, either at 6 months or at 12 months after the beginning of imatinib therapy and response was categorized as Major Molecular Response (MMR) with BCR-ABL1 expression of ≤ 0.1%, and further deep molecular response (MR) as; MR^4^, MR^4.5^ with BCR-ABL1 expression ≤ 0.01% and ≤ 0.0032% respectively compared to the base line levels, and no molecular response (NMR) as defined in methodology section. The molecular response to imatinib with 400 and 800 OD dose arms are shown in Table 7. The rates of major molecular response (MMR) at 6 months and 12 months were 12.66% and 39.33% respectively, while the 6 month and 12- month rates of deeper molecular responses with MR^4^ and MR^4.5^ log reduction were respectively 6.00% vs 5.33% and 15.33% vs 12.00% (Table 7).

**Table 7:** Achievement of molecular response

Time duration After initiation of Treatment (months)	Response	Imatinib 400-mg OD dose group (*n* = 59)	Imatinib 800-mg OD dose group (*n* = 91)	Total proportion of patients (*n* = 150)
6 months	*MMR n(%)	7 (11.86)	12 (13.18)	19 (12.66)
12 months		17 (28.81)	34 (37.36)	51 (34.00)
6 months	*MR^4^ n(%)	4 (6.78)	5 (5.49)	9 (6.00)
12 months		8 (13.56)	14 (15.38)	22 (14.66)
6 months	*MR^4.5^ n(%)	3 (5.08)	5(5.49)	8 (5.33)
12 months		7 (11.86)	11 (12.08)	18 (12.00)

^*^ 23 CML patients failed to achieve molecular response out of which 17 had primary failure and 6 had secondary failure

The patients were then further followed and hematologic assessments (haemoglobin, white blood cells, platelets, percentage of blasts, percentage of eosinophils and percentage of basophils) and presence/absence of hepatosplenomegaly were performed every fifteen days during the course of treatment. The patients were then grouped into three categories (Table 8) according to ELN guidelines[25]; I) Optimal response; associated with long term best outcome, II) Warning; included those patients who required more frequent monitoring so as to allow timely modifications of the therapy in case of developing treatment toxicities and III) Failure; defined those patients who were having stable and increasing disease as evidenced by persistence of CP and progression to advanced phases (AP and BC) of CML disease respectively. Of the 150 patients, 23 (15.33%) patients failed to achieve response to imatinib therapy and of these 23 patients, 17 (11.33%) had primary failure and 6 patients (4.00%) had secondary failure to treatment.

**Table 8:** Categorization of patients on the basis of molecular response

	**OPTIMAL**	**WARNING**	**FAILURE**
No.of patients,n (%)	109 (72.66)	18 (12)	23 (15.33)

### Promoter CpG dinucleotide hypermethylation of apoptosis and cell cycle regulatory genes is associated with disease progression of CML to advanced stages

A total of six genes including apoptosis related genes (DAPK1 and RIZ1), cell cycle regulating genes (p16^INK4A^, RASSF1A and p14^ARF^) and Suppressor of Cytokine Signaling (SOCS1) gene were analysed for promoter CpG dinucleotide methylation in white blood cell DNA from 150 CML patients in different clinical stages of the disease and in matched healthy control subjects by MS-PCR (agarose gel pictures in Fig. 1 of supplementary data). To reveal the methylation pattern of these genes in patients with CML in CP (*n* = 100), AP (*n* = 25) and BC (*n* = 25), fisher’s exact test was applied for comparison between different groups. It was found that the frequency of promoter CpG dinucleotide methylation of all the six genes studied was significantly more in chronic, accelerated and blastic phases in comparison to age and gender matched healthy control subjects.

**Fig. 1:**
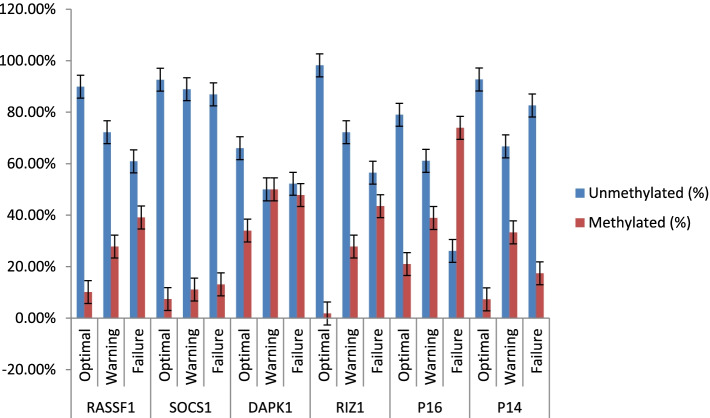
Frequency of CpG dinucleotide hydroxymethylation of different genes among CML patients in different clinical stages of the disease

Moreover, it was observed that frequency of promoter CpG dinucleotide methylation patterns of apoptosis (DAPK1 and RIZ1) and cell cycle related genes (p16^INK4^, RASSF1A and p14^ARF^) was significantly more in advanced stages of CML disease compared to early chronic phase disease. In addition, an increasing promoter CpG dinucleotide methylation frequency was observed in case of SOCS1 gene in accelerated and blastic phases of the CML disease than chronic phase but the differences did not reach statistical significance. A statistical analysis of methylation patterns of the different genes studied and comparison among patients of different clinical stages is described in Table 9 and Fig. 1.

**Table 9:** Promoter CpG dinucleotide methylation of different genes in CML patients and association with different clinical stages

Gene	Stage of disease	Unmethylated n(%)	Methylated n(%)	Chi-Square	*p*-value
RASSF1	CP-CML	94(94%)	6 (6%)	25.87	< 0.0001
	AP-CML	17(68%)	8(32%)		
	BC-CML	14 (56%)	11(44%)		
SOCS1	CP-CML	94 (94%)	6 (6%)	2.95	0.23
	AP-CML	22(88%)	3(12%)		
	BC-CML	21(84%)	4(16%)		
DAPK1	CP-CML	71(71%)	29(29%)	11.67	0.003
	AP-CML	13 (52%)	12(48%)		
	BC-CML	9 (36%)	16(64%)		
RIZ1	CP-CML	93 (93%)	7(7%)	6.40	0.04
	AP-CML	21(84%)	4(16%)		
	BC-CML	19 (76%)	6(24%)		
P16INK4A	CP-CML	79(79%)	21(21%)	18.23	0.0001
	AP-CML	15(60%)	10(40%)		
	BC-CML	9(36%)	16(64%)		
P14ARF	CP-CML	94(94%)	6(6%)	10.98	0.004
	AP-CML	18(72%)	7(28%)		
	BC-CML	20(80%)	5(20%)

### Promoter CpG hypermethylation of apoptosis and cell cycle regulatory genes is associated with poor molecular response in CML patients

**Fig. 2:**
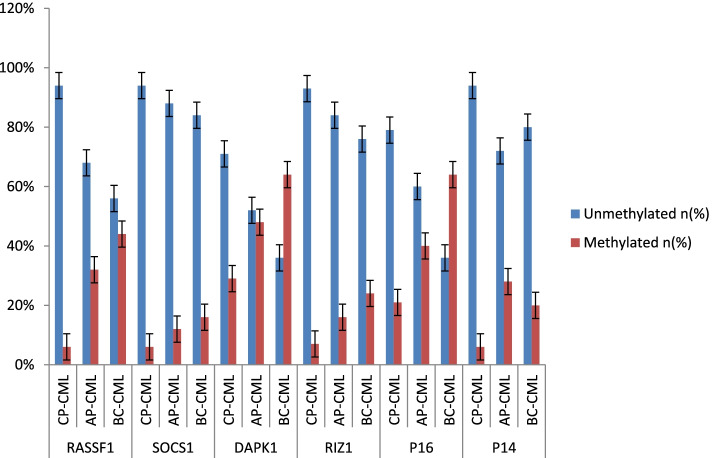
Frequency of CpG dinucleotide hydroxymethylation of different genes among CML patients in different imatinib response categories

Next hydroxymethylation of promoter CpG dinucleotides of the six genes studied was analysed among patients grouped on the basis of their response to imatinib therapy. It was found that promoter hypermethylation of RASSF1, p16^INK4^ and p14^ARF^ and RIZ-1 genes was a characteristic feature of patients in warning and failure groups as the proportion of patients, with promoter CpG dinucleotide hydroxymethylation of these genes, was found to be significantly more in imatinib failure and warning groups as compared to optimal response group. The proportion of patients, with promoter CpG dinucleotide methylation of DAPK1 (an apoptosis related) gene, was observed to be more in warning group (50%) followed by imatinib failure group (47.8%) and then by optimal response category (34.0%), but the difference was not statistically significant (*p* = 0.24). Further, the corresponding proportion of patients with promoter CpG dinucleotide methylation of cytokine signalling gene SOCS1 was 7.4%, 11.1% and 13.1% in optimal, warning and failure groups, however, this progressive increase in proportion of patients with SOCS1 promoter CpG dinucleotide hydroxymethylation from optimal response category to warning and failure did not reach any statistical significance. The association of molecular response of CML patients to imatinib and promoter CpG dinucleotide hydroxymethylation is depicted in Table 10 and Fig. 2.

**Table 10:** Promoter CpG dinucleotide methylation of different genes in CML patients and association with molecular response

Gene	Response	Unmethylated (%)	Methylated (%)	Chi-Square	*p*-value
RASSF1	Optimal	98 (89.9%)	11 (10.1%)	13.35	0.001
	Warning	13(72.2%)	5 (27.8%)		
	Failure	14 (60.9%)	9 (39.1%)		
SOCS1	Optimal	101(92.6%)	8(7.4%)	0.94	0.62
	Warning	16(88.9%)	2(11.1%)		
	Failure	20(86.9%)	3(13.1%)		
DAPK1	Optimal	72 (66.0%)	37(34.0%)	2.80	0.24
	Warning	9(50.0%)	9(50.0%)		
	Failure	12(52.2%)	11(47.8%)		
RIZ1	Optimal	107(98.2%)	2(1.8%)	38.28	< 0.001
	Warning	13(72.2%)	5(27.8%)		
	Failure	13(56.5%)	10(43.5%)		
P16INK4A	Optimal	86(79.0%)	23(21.0%)	25.16	< 0.0001
	Warning	11(61.1%)	7(38.9%)		
	Failure	6(26.1%)	17(73.9%)		
P14ARF	Optimal	101(92.7%)	8(7.3%)	10.63	0.005
	Warning	12(66.7%)	6(33.3%)		
	Failure	19(82.6%)	4(17.4%)

### Kaplan–meier analysis for overall survival related to hydroxymethylation of various genes

**Fig. 3:**
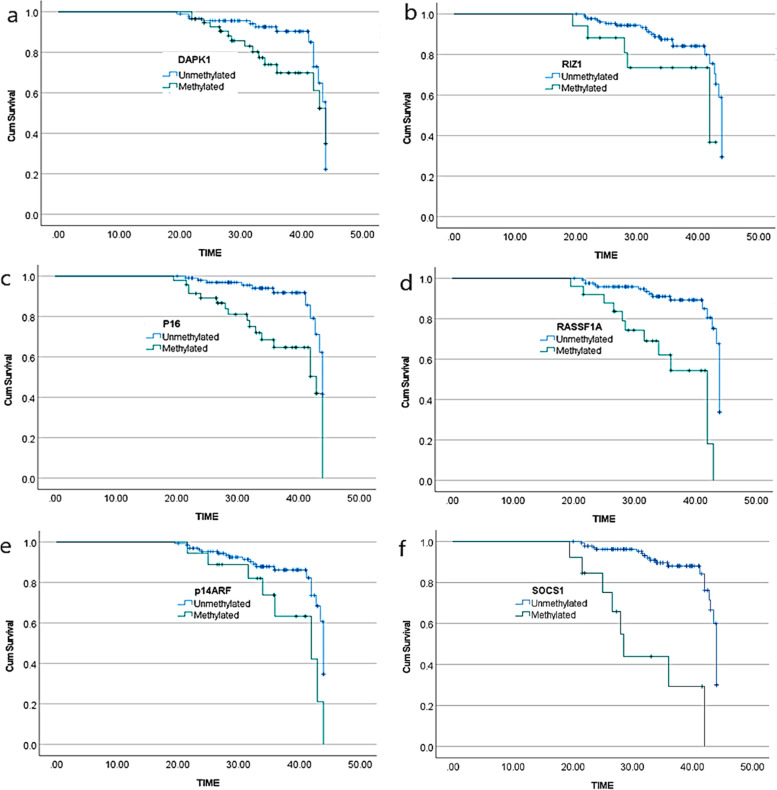
Survival analysis in concert with promoter hydroxymethylation of cell cycle regulating and apoptosis related genes in CML patients

After a follow-up of 48 months, estimated overall survival for CpG dinucleotide methylation of the studied genes was analysed. This was observed that overall survival significantly differed in patients with hypermethylation as compared to those with hypomethylation of cell cycle regulated genes: p16^INK4A^ (median survival for hypomethylation = 44 moths; CI = 43.30–44.69 and median survival for hypermethylation = 43 months; CI = 35.80–50.20; *p* = 0.0001), RASSF1 (median survival for hypomethylation = 44 months; CI = 43.62–44.36 and median survival for hypermethylation = 42 months; CI = 36.53–47.46; *p* = 0.0001) and p14^ARF^ (median survival for hypomethylation = 44 months; CI = 43.51–44.48 and median survival for hypermethylation = 42 months; CI = 31.09–52.90; *p* = 0.01). Also, hypomethylation of Suppressor of Cytokine Signalling (SOCS1) gene was found to be associated with better survival (median survival for hypomethylation = 44 months; CI = 43.61–44.38 and median survival for hypermethylation = 28 months; CI = 25.22–31.17; *p* < 0.0001). However, promoter methylation of apoptosis related genes including DAPK1 and RIZ1 did not exhibit any significant differences in overall survival of patients with hypo and hypermethylation status (*p* = 0.09; *p* = 0.05 respectively). The results are depicted in Fig. 3.

## Discussion

The mechanisms which contribute to progression of CML disease and drug response vary to a considerable extent and have not been clearly understood. The various events that have, till now, been found as culprits for the progression of CML to advanced clinical stages and drug response may be categorized at cellular and molecular levels. The culprits at cellular level include increased proliferation, decreased apoptosis, differentiation halt, abnormal immune surveillance while activation of oncogenes, tumor suppressor gene inactivation, genomic instability, impaired DNA repair mechanisms among others are the molecular culprits [28]. The control of most of the above cited events can be attributed to changes in the genes that are at the heart of that particular event. Apoptosis, differentiation, DNA repair for example are controlled by the activity of genes related to these processes and among the various mechanisms, DNA methylation in the promoter regions of a gene is one of the important processes that controls gene expression. DNA hyper and hypomethylation are respectively associated with decreased and ectopic expression of the genes. Moreover, DNA methylation at CpG dinucleotides could also influence differential promoter usage influencing gene expression patterns [29].

To find the association of methylation status of the genes with CML disease progression and imatinib drug responses, we analysed the methylation differences among CML patients in different clinical stages and among age and gender matched healthy controls. We report here that CML patients possessed significant hypermethylation in all the genes studied, in comparison to healthy control subjects. It was also observed that methylation at promoter CpG dinucleotide regions of cell cycle regulating genes including RASSF1, p16^INK4A^ and p14^ARF^ and apoptosis related genes DAPK1 and RIZ-1 are characteristics of poor respondents of imatinib drug. We found significant association of increased methylation patterns of the above cited genes with poor imatinib response as judged by the proportion of patients with hypermethylation of the genes to be more in warning and failure groups as compared to optimal response group. Moreover, the proportion of CML patients with hypermethylation of promoter CpG dinucleotides of DAPK1, RIZ1, P16INK4A, RASSF1A and p14ARF^ARF^ genes was significantly more in advanced disease (AP and BC) stages in comparison to early CML (CP) disease subjects. These findings of our current study confirm and extend the reports of previous studies [30–33].

Methylation of cell cycle regulating genes like p16^INK4A^ has been found to be associated with progression of CML disease and is reported to be associated with late stage CML disease in other studies [33, 34]. Moreover, progression to lymphoid blast crisis has been found to be associated with homozygous deletions of p16^INK4A^ gene [34, 35]. RASSF1 promoter methylation has been seen in CML-derived erythroleukaemia K562 cell line but the results could not be replicated in CML patients at different clinical stages [36]. However, this is not in accordance to our study, we report that RASSF1 methylation is associated with CML disease progression based on the finding that proportion of patients with RASSF1 methylation in advanced AP and BC CML disease was more than that of CP disease. This contradiction in our results to those of Avramouli A et al., 2009, might be because of smaller number of patients (*n* = 31) included in the later study [36]. One of the frequently altered cell cycle regulating genes is p14^ARF^ which has been reported to be inactivated through various mechanisms viz, mutations, deletions and DNA methylation in variety of malignancies of diverse origin [37]. p16^INK4A^ and p14^ARF^ inactivation through promoter methylation are reported to be important events associated with accelerated phase of CML disease [38]. However, the results need to be validated in a large cohort of CML patients in all the three clinical stages since the total number of subjects in which the reports have been investigated by E Nagy et al., 2003, was too small (*n* = 30). In this study, the same results have been replicated in comparatively large number of subjects. Further, we have previously found that methylation of p16^INK4A^ gene is one of the primary events in CML disease progression [39].

The epigenetic changes of apoptosis related genes are reported to be associated with progression of both solid tumors and hematologic malignancies as well [40]. Promoter methylation of DAPK1, for example, is a characteristic feature of breast cancer [41]. In this study, the promoter methylation of DAPK1 was found to be related to accelerated phase and blast crisis. However, it is reported that promoter methylation of RIZ1, another apoptosis related gene, is not a characteristic feature of advanced CML disease which is in contrast to present findings which could again be attributed to the smaller number of subjects included in the previously reported study [31]. RIZ1 gene inactivation during blast crisis occurs through epigenetic silencing and has been suggested as a predictive marker for imatinib resistance and CML disease progression [42]. Reduced expression of RIZ1 and DAPK1 due to promoter hypermethylation has been reported in other malignancies as well, such as cervical cancer/ cervical neoplasia [43, 44], thyroid tumorigenesis [45], stomach carcinogenesis [46], cervical cancer [43], lung cancer patients[47–49]. Therefore, the above discussion of promoter methylation and disease progression of CML to advanced phases supports the idea of considering the use of epigenetic drugs along with tyrosine kinase inhibitor (TKI) therapy. This may help a significant number of CML patients in better management of the disease.

The identification of methylation of apoptosis related DAPK1 and RIZ1 genes in concert with drug response and prognosis is utmost important. We observed that DAPK1 and RIZ1 promoter methylation is significantly associated with poor imatinib response in CML patients. Methylation of DAPK1 is reported in other cancers like gastric cancer [50–54]. There are contradictory reports regarding methylation of DAPK1 in concert with drug response and prognosis for example no correlation of DAPK1 methylation was found with prognosis in ovarian cancer [55] and non small cell lung cancer[56]. However, there are reports suggesting a strong association of DAPK1 hypermethylation with poor disease specific survival and therapy response [57]. Hypermethylation of RIZ1 has been reported for its inactivation and silencing [58]. In one of the studies from our lab, we have reported that RIZ1 promoter methylation increases progressively with advanced CML disease stages and that its expression may be a cause, among others, for poor drug response [59]. In another study, we have reported that decrease in RIZ1 gene is responsible for increased IGF1 expression in K562 CML blast crisis cell line and in advanced disease CML patients [60]. Yet another report from our lab has observed that inactivation of RIZ1 gene by insertion/deletion polymorphism and promoter hypermethylation is associated with CML disease progression and imatinib resistance [61]. In the present study, we observed that promoter hypermethylation of RIZ1 is significantly more frequent in advanced CML disease compared to early disease and is a characteristic feature of poor imatinib respondents. But we could not find any statistical difference in proportion of patients having hyper and hypomethylation of RIZ1 gene promoter in relation to overall survival. RIZ1 reduced expression has been reported in other haematological malignancies [62]. In adult acute lymphoblastic leukaemia, reduced RIZ1 gene expression has been found to be associated with leukemogenesis. Inactivation of RIZ1 is a characteristic feature of T-ALL [62]. However, further studies are required for elucidation of the inactivation mode of RIZ1 and its intricate role in development and progression of different types of malignancies and drug response.

RASSF1A promoter methylation is speculated to influence drug sensitivity of tumors like non small cell lung carcinoma [63], esophageal squamous carcinoma tumorigenesis [64], breast cancer patients [65]. In addition, there are reports suggesting utilization of RASSF1A methylation patterns for monitoring response to adjuvant therapy in the clinic, as RASSF1A methylation depletion has been found to be linked with good response to adjuvant regimens[66]. Apparent methylation patterns of RASSF1A gene are reported as biomarkers of lung cancer diagnosis, treatment and prognosis[67]. RASSF1A and its epigenetics have gained much attention due to its increasing occurrence in diverse cancer types. Promoter methylation of RASSF1A, which is preceded by histone modifications, has been reported as an epigenetic candidate marker in a variety of cancers with diverse origin. There are reports which suggest that its epigenetic abrogation may promote expression of RASSF1C which is a putative oncogenic isoform [68]. However, some studies discuss RASSF1A methylation in non small cell lung carcinoma and associate it with good response [69]. Therefore, a better understanding of the significance of RASSF1A methylation patterns in various cancer types becomes imperative for its clinical and drug behavior role. Our results indicate that RASSF1A hypermethylation characterizes poor imatinib response and poor survival of CML patients treated with imatinib.

p16^INK4a^ expression has been found to be associated with poor prognosis in ER-positive, PR-negative and HER2- negative tumors and hence reported as a predictive prognostic indicator to predict treatment response for hormonal therapy [70]. Hypermethylation of p16^INK4A^ and p14^ARF^ has been suggested to possess predictive properties for a variety of clinicopathological outcomes. Moreover, p14^ARF^ and p16^INK4A^ gene inactivation has been reported in development of colon carcinoma [71], cervical cancer [72], hematological malignancies[73]. It is suggested that methylation profile of p14^ARF^ and p16^INK4A^ might be playing an important role in distinct subsets of colon carcinoma[74]. The observation from our present study that hypermethylation of these two genes accumulate in patients with poor drug response and poor overall survival and the reports from previous studies discussed above indicate that methylation status of p16^INK4A^ and p14^ARF^ can definitely be used as promising candidate predictors of response to therapy and clinical outcome.

Suppressor of cytokine signaling-1 (SOCS1) gene has been recognised as tumor suppressor gene and found to be related to lymphatic metastasis and disease progression of liver cancer [75]. Silencing of SOCS1 by methylation is reported in hepatocellular carcinoma and other tumors like cervical cancer [76], hepatoblastoma[77], esophageal squamous cancers[78], melanoma, squamous cell carcinoma of the head and neck, pancreatic carcinoma and breast and ovarian cancer [79]. SOCS1 gene methylation has been reported to cause gene silencing which is accompanied by downstream JAK/STAT signaling and promotion of cell proliferation in acute myeloid leukaemia [80]. In this study, although we found a higher proportion of patients with SOCS1 methylation in advanced disease stages and poor imatinib respondents, but the difference was not statistically significant. Our results are slightly different from the previous study by Ta Chih Liu et al. (2003) [81] which reports that SOCS1 gene methylation plays an important role in the pathogenesis of CML disease progression. This discrepancy might be attributed to the environmental effects and ethnicity of the cases being studied. The methylation status of the DNA and histone proteins depends on ethnicity of population [82]. However, we did observe that hydroxyrmethylation of SOCS1 gene was significantly associated with poor overall survival of CML patients on imatinib therapy. Therefore, we suggest that there is need of more studies to conduct for studying the exact role of SOCS1 methylation and expression patterns that will provide more detailed insight of the role of SOCS1 methylation in CML disease progression and imatinib response. The major limitation of our study was that we did not characterize the methylation pattern of the genes quantitatively. Another limitation of our study was that we were not able to include homogenous number of patients in each clinical stage of the disease.

Based on the present study and from the above discussion, it can be inferred that hydroxymethylation of DAPK1, RIZ1, P16INK4A, RASSF1A and p14ARF^ARF^ are the main characteristics of a variety of malignancies including haematological ones. Therefore, more accurate and site specific methylation patterns of genomic loci should be focused in future studies. These studies would benefit to identify the methylation patterns of the genes involved in cancer development, progression and prognosis and illustrate the feasibility of epigenome target therapy. Moreover, epigenome insights underlying a disease development/progression will help in designing personalized therapy [83].

## Conclusion

The results of this study indicate that hydroxymethylation of promoter regions of DAPK1, RIZ1, P16INK4A, RASSF1A and p14ARF^ARF^ genes may be an important marker of CML disease progression, defines poor imatinib respondents and characterizes poor overall clinical outcome of CML patients on imatinib therapy. Hence, our study supports the rationale of using demethylating agents in combination with tyrosine kinase inhibitor therapy for better clinical outcome.

## Supplementary Information

**Additional file 1: Figure 1. **MS-PCRagarose gel pictures for different genes studied.

## Data Availability

All the data generated and analysed in this study has been provided in this published manuscript and its supplementary information files.

## Abbreviations

CML: Chronic Myeloid Leukaemia
Ph Chromosome: Philadelphia Chromosome
TKIs: Tyrosine Kinase Inhibitors
BCR: Breakpoint Cluster Region
ABL: Abelson Leukaemia Viral Oncogene
SOCS1: Suppressor of Cytokine Signalling 1
RIZ1: Reinoblastoma protein-interacting zinc-finger gene 1
p14^ARF^: ARF tumor suppressor
p16^INK4a^: Cyclin Dependent kinase inhibitor 2A
DAPK1: Death Associated Protein Kinase 1
