# Correction: Aberrant hydroxymethylation in promoter CpG regions of genes related to the cell cycle and apoptosis characterizes advanced chronic myeloid leukemia disease, poor imatinib respondents and poor survival

**DOI:** 10.1186/s12885-022-09568-3

**Published:** 2022-04-22

**Authors:** Sameer Ahmad Guru, Mamta Pervin Sumi, Rashid Mir, Mirza Masroor Ali Beg, Bidhan Chandra koner, Alpana Saxena

**Affiliations:** 1 Lurie Children’s Hospital and Stanley Manne Children’s Research Institute, Northwestern University, Chicago, IL USA; 2 Department of Biochemistry, Multidisciplinary Research Unit (MRU), Maulana Azad Medical College, New Delhi, India; 3 Department of Inflammation and Immunity, Lerner Research Institute, Cleve Land Clinic OH, Cleveland, USA; 4 Kingdom of Saudi Arabia, University of Tabuk, Tabuk, Saudi Arabia; 5 Faculty of Medicine and Center for Promotion of Medical Research, Faculty of Medical Sciences, Ala-Too International University, Bishek, Kyrgyzstan; 6 Department of Biochemistry, Hamdard Institute of Medical Science and Research (HIMSR), New Delhi, India

**Correction to: BMC Cancer 22, 1-15 (2022)**

**https://doi.org/10.1186/s12885-022-09481-9**

Following publication of the original article [1], the authors identified an error in the author name of Rashid Mir.

The incorrect author name is: Abdul Rashid Mir

The correct author name is: Rashid Mir

The author group has been updated above and the original article [1] has been corrected.
