# Supplementary material for: Aberrant hydroxymethylation in promoter CpG regions of genes related to the cell cycle and apoptosis characterizes advanced chronic myeloid leukemia disease, poor imatinib respondents and poor survival
Source: BMC Cancer. 2022 Apr 14;22:405. doi: 10.1186/s12885-022-09481-9 (PMC9008925; doi:10.1186/s12885-022-09481-9)
Supplement: Supplementary file 1 — Additional file 1: Figure 1. MS-PCRagarose gel pictures for different genes studied. [file 12885_2022_9481_MOESM1_ESM.docx]

**Supplementary Materials:**

Figure 1: **MS-PCR agarose gel pictures for different genes studied:**

**(***Representative data, U; unmethylation, M; methylation, L; 100bp DNA Ladder)***.**

1. **DAPK1 promoter gene methylation**

**M**

**Patient 1** and patient 3 are methylated.

Patient 2 is unmethylated.

1. **RIZ1 promoter gene methylation:**


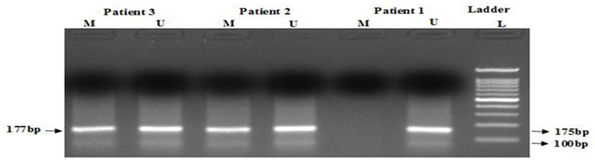


Patient 1 is unmethylated.

Patient 2 and patient 3 are methylated.

1. **P16 promoter gene methylation:**

**P1**. Patient 1 and patient 2 are unmethylated.

Patient 3 is methylated.

1. **RASSF1 promoter gene methylation:**

Patient 1 is unmethylated.

Patient 2 and patient 3 are methylated.

1. **P14 promoter gene methylation:**

Patient 1 and Patient 2 are unmethylated.

Patient 3 is methylated.

1. **P14 promoter gene methylation:**

Patient 1 and Patient 2 are unmethylated.

patient 3 is methylated.
